# Inference of Intercellular Communications and Multilayer Gene-Regulations of Epithelial–Mesenchymal Transition From Single-Cell Transcriptomic Data

**DOI:** 10.3389/fgene.2020.604585

**Published:** 2021-01-08

**Authors:** Yutong Sha, Shuxiong Wang, Federico Bocci, Peijie Zhou, Qing Nie

**Affiliations:** ^1^Department of Mathematics, University of California, Irvine, Irvine, CA, United States; ^2^The NSF-Simons Center for Multiscale Cell Fate Research, University of California, Irvine, Irvine, CA, United States; ^3^Department of Developmental and Cell Biology, University of California, Irvine, Irvine, CA, United States

**Keywords:** single-cell RNA sequencing, trajectory inference, gene regulatory network, cell fate decision, cell–cell communication, multi-scale analysis

## Abstract

Epithelial-to-mesenchymal transition (EMT) plays an important role in many biological processes during development and cancer. The advent of single-cell transcriptome sequencing techniques allows the dissection of dynamical details underlying EMT with unprecedented resolution. Despite several single-cell data analysis on EMT, how cell communicates and regulates dynamics along the EMT trajectory remains elusive. Using single-cell transcriptomic datasets, here we infer the cell–cell communications and the multilayer gene–gene regulation networks to analyze and visualize the complex cellular crosstalk and the underlying gene regulatory dynamics along EMT. Combining with trajectory analysis, our approach reveals the existence of multiple intermediate cell states (ICSs) with hybrid epithelial and mesenchymal features. Analyses on the time-series datasets from cancer cell lines with different inducing factors show that the induced EMTs are context-specific: the EMT induced by transforming growth factor B1 (TGFB1) is synchronous, whereas the EMTs induced by epidermal growth factor and tumor necrosis factor are asynchronous, and the responses of TGF-β pathway in terms of gene expression regulations are heterogeneous under different treatments or among various cell states. Meanwhile, network topology analysis suggests that the ICSs during EMT serve as the signaling in cellular communication under different conditions. Interestingly, our analysis of a mouse skin squamous cell carcinoma dataset also suggests regardless of the significant discrepancy in concrete genes between *in vitro* and *in vivo* EMT systems, the ICSs play dominant role in the TGF-β signaling crosstalk. Overall, our approach reveals the multiscale mechanisms coupling cell–cell communications and gene–gene regulations responsible for complex cell-state transitions.

## Introduction

Epithelial-to-mesenchymal transition (EMT) is a biological process where epithelial cells lose cell–cell adhesion and gain some mesenchymal traits of migration and invasion (Kalluri and Weinberg, 2009; Jolly et al., 2018). EMT not only occurs widely during normal embryonic development, organ fibrosis, and wound healing, but also plays an important role in tumor progression with metastasis (Nieto et al., 2016; Lambert et al., 2017).

Recent studies have underscored that EMT is not a binary process, but instead exists on a spectrum with various hybrid states ranging from epithelial-to-mesenchymal phenotypes (Nieto et al., 2016). Cells undergoing EMT can display mixed epithelial and mesenchymal features and are considered in the intermediate cell states (ICSs; Jolly et al., 2015; Sha et al., 2019; Jia D. et al., 2019). In the context of cancer progression, these ICSs have been proposed as the main drivers of metastasis because of their ability to undergo collective cell migration as highly metastatic multicellular clusters (Jolly et al., 2015). Therefore, understanding the features and role of ICSs during EMT could potentially unlock novel clinical strategies. With the unprecedented opportunities brought by single-cell RNA sequencing (scRNA-seq), the existence of multiple ICSs and their transcriptomic profiles has been observed and analyzed via pseudotemporal ordering or energy landscapes (Qiu et al., 2017; Jin et al., 2018; Li and Balazsi, 2018; Pastushenko et al., 2018; An et al., 2019; Chen et al., 2019). Very recently, specially designed methods have also been proposed to infer EMT trajectories or transition paths from the single-cell transcriptomic (Sha et al., 2020) or imaging data (Wang W. et al., 2020). The integrative analysis combining unsupervised learning of single-cell transcriptomic data and computational modeling of EMT in cancer and embryogenesis successfully uncovered the novel roles of ICSs on adaption, noise attenuation, and transition efficiency (Sha et al., 2020). While these methods have provided insights into the dynamics of EMT from a single-cell perspective, the role of intercellular communication in EMT remains largely unknown.

Indeed, EMT is not necessarily a cell autonomous process. Cells secrete and in turn respond to various growth and differentiation signaling factors secreted by other cells in the extracellular environment, including transforming growth factor β (TGF-β), WNT, and Notch proteins (Moustakas and Heldin, 2007; Xu et al., 2009; Boareto et al., 2016; Bocci et al., 2018). Among them, the well-characterized TGF-β pathway has received much attention as a major inducer of EMT during embryogenesis, cancer progression, and fibrosis (Wendt et al., 2009; Xu et al., 2009). The TGF-β pathway can also crosstalk with other pathways such as WNT and SHH (Zhang et al., 2016), forming the complex response of signaling. In addition, signaling in cell–cell communications has also been found important in the formation and regulation of ICSs (e.g., through Notch pathway; Bocci et al., 2020). This intercellular communication has been shown to play significant roles in regulating gene expression dynamics within individual cells, through analysis of scRNA-seq datasets from several development and cancer systems (Camp et al., 2017; Puram et al., 2017; Zepp et al., 2017; Kumar et al., 2018; Wang S. et al., 2020). Computational methods have been developed to infer cell–cell communication networks based on ligand–receptor interactions (Wang S. et al., 2019; Wang Y. et al., 2019; Cabello-Aguilar et al., 2020; Jin et al., 2020) and elucidate how cell–cell communications propagate to downstream target genes through transcription factors (Browaeys et al., 2020). While methods have been developed to infer EMT gene regulatory network (GRN) from RNA-seq single-cell data (Ramirez et al., 2020), the role of cell–cell communications on gene regulation dynamics along EMT trajectory is poorly understood.

Through both experimental and mathematical modeling studies, the key circuits of EMT involving few epithelial/mesenchymal markers, transcription factors, and signaling molecules have been summarized (Hong et al., 2015; Li et al., 2016; Fazilaty et al., 2019; Kang et al., 2019; Xing and Tian, 2019; Tripathi et al., 2020; Yang et al., 2020). Because of different roles of nodes, the circuits can be modeled as a multilayer network (Kivelä et al., 2014) with hierarchical structures (Browaeys et al., 2020). In the multilayer network, cells communicate with each other and the environment via signal transduction pathways (Layer 1), which directly targets the downstream factors or genes (Layer 2), that subsequently regulate the expression of marker genes of various cell states (Layer 3). In addition, there may be dynamical changes of network structure during EMT, where the temporal (or pseudotemporal) information constitutes another independent dimension of the layer sets. The complex interactions among nodes may exist within the same layers or across different layers, in controlling EMT.

Here we study the time-series scRNA-seq datasets of OVCA420 cancer cell line exposed to various EMT-inducing factors (Cook and Vanderhyden, 2020). We first delineate the underlying transition details at individual cell resolution with a recently developed method, QuanTC. For the cancer cell lines undergoing EMT under three different treatments, we quantify the ICS-regulated trajectories and detect the driver genes in EMT for each case, respectively. While cells undergo TGFb1-driven EMT in a highly synchronized fashion, EMT guided by epidermal growth factor (EGF) and tumor necrosis factor (TNF) is asynchronous. Next, we develop a multilayer network approach to infer and visualize the hierarchical interactions that combine cell–cell communications through the TGF-β pathway, signal transductions, and GRNs from single-cell transcriptomic data. After trajectory inference, we then utilize the multilayer network approach to decipher the role of TGF-β pathway in regulating EMT dynamics with different inducing factors. We also compare the results of *in vitro* cancer cell lines with further analysis of *in vivo* mouse skin squamous cell carcinoma (SCC) dataset (Pastushenko et al., 2018).

## Results

### Synchronous EMT With Two ICSs Induced by TGFB1

We analyzed the published datasets (Cook and Vanderhyden, 2020) with ovarian OVCA420 cancer cell line capable of undergoing EMT. This cell line, which normally shows an epithelial morphology, was exposed to known EMT-inducing factors: TGFB1, EGF, and TNF, respectively, to promote EMT. We used the samples collected at five distinct time points from day 0 to day 7 after the treatment.

**FIGURE 1:**
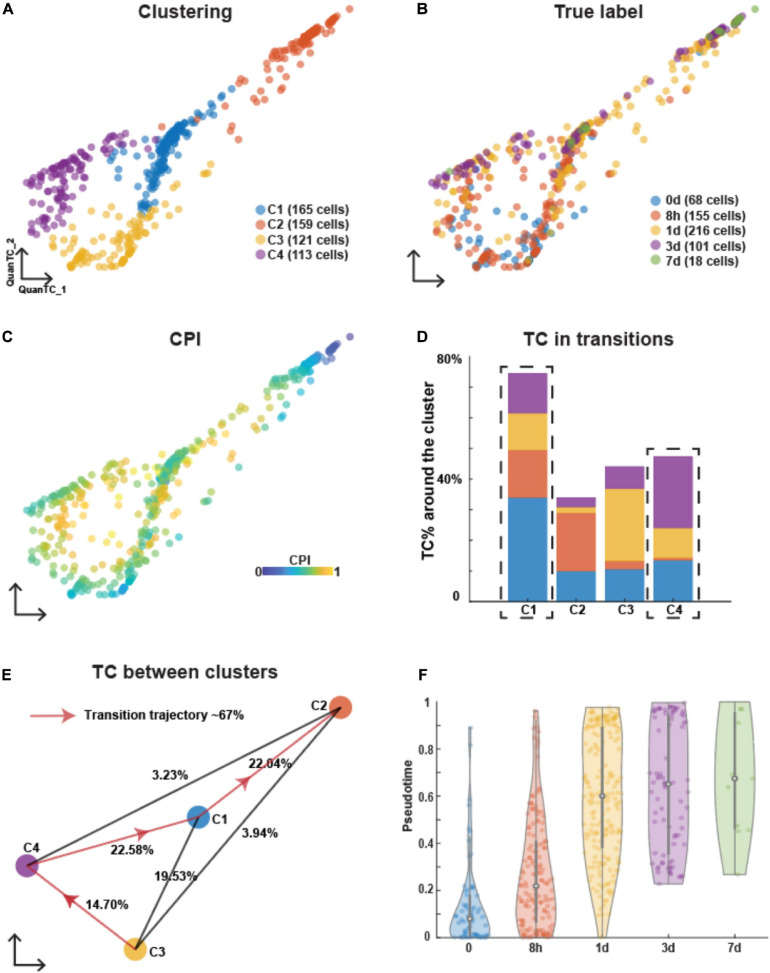
Analyzing OVCA420 cancer cell line undergoing EMT induced by TGFB1 using QuanTC. **(A–C)** Visualization of cells in the two dimensional space by QuanTC. Each circle represents one cell colored by clustering **(A)**, the collection time of the samples after the treatment **(B)**, and CPI values **(C)**. **(D)** Percentage of TC associated with each cluster relative to the total number of TC. The dashed box covers the ICS having more TC around. The parameters to choose TC are given in Supplementary Table 1. **(E)** Visualization of cluster centers with color consistent with **(A)**. Each percentage on the line show the percentage of TC between two clusters relative to the total number of cells. Arrowed solid line shows the main transition trajectory. **(F)** Violin plot of pseudotime value of each cell vs the collection time points. Each dot represents a cell colored by collection time points. The circle displays the mean and vertical line shows the interquartile ranges.

To compare the process of EMT under three treatments, we used QuanTC (Sha et al., 2020) to perform the clustering and transition trajectory reconstruction. QuanTC estimates the optimal number of clusters by analyzing the sorted eigenvalues of symmetric normalized graph Laplacian (Supplementary Figure 1A). Four clusters were identified in EMT induced by TGFB1 (Figure 1A). A first cluster (C3) was mostly composed by cell subpopulations collected at day 0 and 8 h after induction (Figure 1B) and expressed relatively high levels of epithelial markers CDH1 (Supplementary Figure 1B). Conversely, a second cluster (C2) consisted of cells collected at days 3 and 7 (Figures 1A,B) and expressed relatively high levels of mesenchymal markers FN1 and SNAI2 (Supplementary Figure 1C). Furthermore, cells in these clusters had a low Cell Plasticity Index (CPI). CPI employs an entropy-based approach to estimate cell plasticity, so that a higher index implies a higher probability of transition between clusters (see section “*Materials and Methods*”). Based on the CPI values, QuanTC predicted that clusters C2 and C3 have lower percentages of transition cells (TCs; Figures 1C,D), thus suggesting that they are the beginning or end of the trajectory. Based on these observations, we identified cluster C3 as the E state and cluster C2 as the M state.

After choosing the E state, C3, as the beginning of the transition, QuanTC computed the most probable transition trajectory, C3–C4–C1–C2, consisting of 67% of the total cell population (Figure 1E). The cluster C4 and C1 were thus identified as ICSs I1 and I2, respectively. The marker genes of each state and the transition genes marking the transition between states along the transition trajectory were inferred by QuanTC (Supplementary Figure 1D). To characterize the two ICSs, I1 and I2, we performed a Gene Ontology (GO) biological processes analysis (The Gene and Ontology Consortium, 2019) of the top 50 marker genes of each state (Supplementary Figure 1E). Both ICSs shared similar biological processes including signaling and localization. Furthermore, I2 also related to adhesion and locomotion. This suggested that the cells in ICSs displayed both epithelial and mesenchymal features and communications with other cells through cell signaling.

Finally, we inspected the population dynamics during TGFB1-driven EMT by considering the pseudotime distribution. Pseudotime quantifies the position of a given cell along the transition trajectory predicted by QuanTC and therefore does not necessarily correlate with the experiment’s physical time. In this time series, however, most cells at *t* = 0 days were characterized by a low pseudotime (i.e., they were positioned toward the beginning of the transition trajectory), whereas cells at later time points exhibited progressively higher pseudotime values (Figure 1F). In other words, OVCA420 cells started from the E state and progressively transitioned throughout the 7 days of EMT induced by TGFB1 in a nearly synchronous fashion.

### Asynchronous EMT Induced by EGF and TNF

**FIGURE 2:**
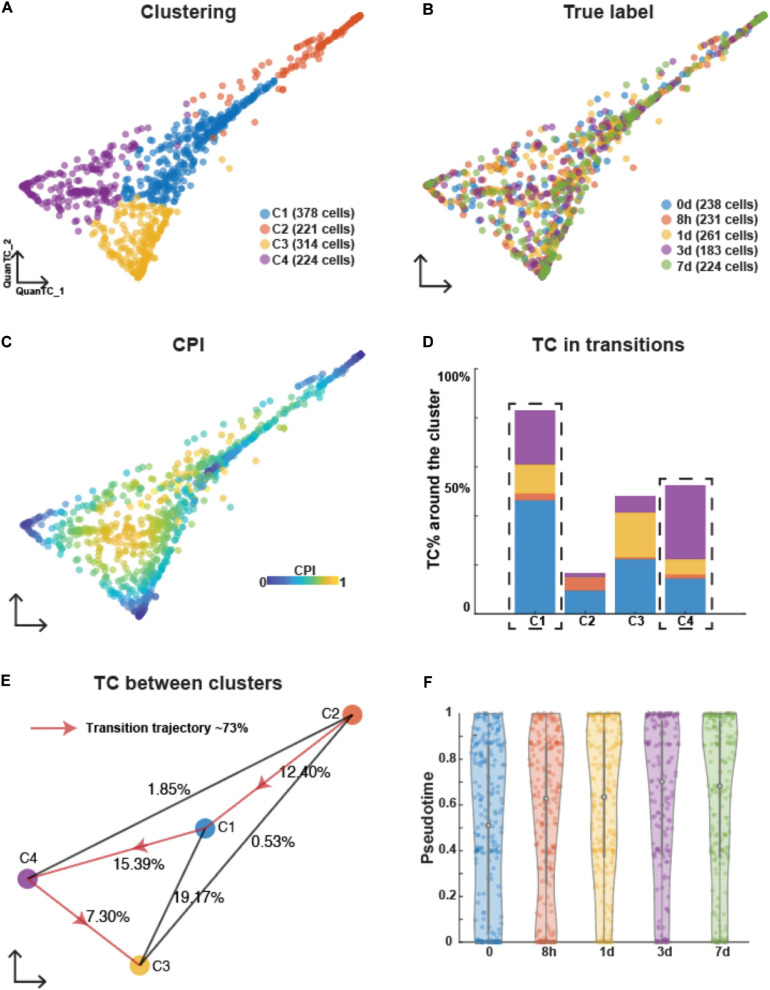
Analyzing OVCA420 cancer cell line undergoing EMT induced by EGF using QuanTC. **(A–C)** Visualization of cells. Each circle represents one cell colored by clustering **(A)**, the collection time of the samples after the treatment **(B)**, and CPI values **(C)**. **(D)** Percentage of TC associated with each cluster relative to the total number of TC. The dashed box covers the ICS having more TC around. The parameters to choose TC are given in Supplementary Table 1. **(E)** Visualization of cluster centers with color consistent with **(A)**. Each percentage on the line show the percentage of TC between two clusters relative to the total number of cells. Arrowed solid line shows the main transition trajectory. **(F)** Violin plot of pseudotime value of each cell vs the collection time points. Each dot represents a cell colored by collection time points. The circle displays the mean and vertical line shows the interquartile ranges.

Applying QuanTC to the OVCA420 dataset where EMT was induced by EGF, four clusters were also identified based on the biggest eigenvalue gap after the first two eigenvalues because we want to investigate the ICSs during EMT (Supplementary Figure 2A and Figure 2A). Differently from TGFB1-driven EMT, however, cells collected at different time points colocalized within the same clusters, and no group of cells at any given time point dominated any cluster (Figure 2B). Based on the CPI values, the two clusters (C2 and C3) were considered as the E and M states based on the fewer TCs around them (Figures 2C,D). Specifically, C2 was then identified as the E state according to the relatively high expression levels of epithelial markers CDH1 (Supplementary Figure 2B), and C3 was identified as the M state because of higher expressions of mesenchymal markers FOXC2 and SNAI2 (Supplementary Figure 2C).

The most probable transition trajectory was inferred after choosing cluster C2 as the starting state (Figure 2E). The two remaining clusters (C1 and C4) between E and M along the transition trajectory had more TCs around them and were identified as I1 and I2, respectively. According to the GO analysis of the top marker genes (Supplementary Figure 2D), the I2 state displayed biological processes including adhesion, locomotion, and signaling, showing mixed feature of both epithelial and mesenchymal cells (Supplementary Figure 2E).

The average pseudotime values slightly increased along collection time points, hence demonstrating that the EGF stimulus induces an EMT response. Compared to TGFB1-driven EMT, however, pseudotime distribution within each time point had a high variance, thus indicating that the EMT induced by EGF was more asynchronous (Figure 2F).

**FIGURE 3:**
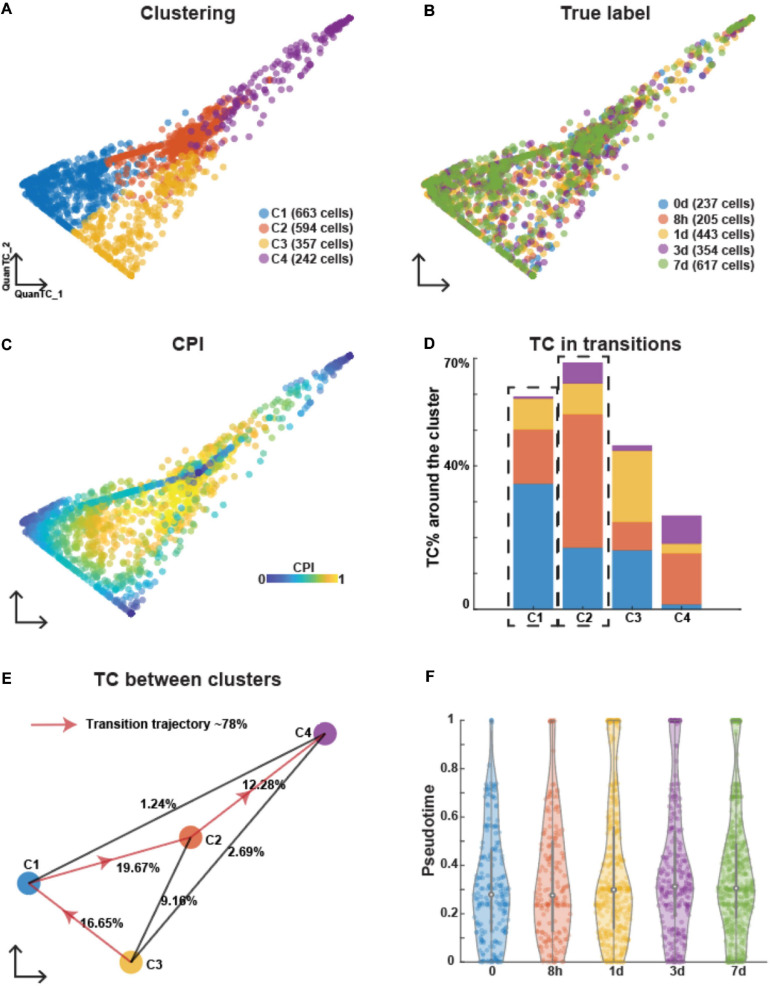
Analyzing OVCA420 cancer cell line undergoing EMT induced by TNF using QuanTC. **(A–C)** Visualization of cells. Each circle represents one cell colored by clustering **(A)**, the collection time of the samples after the treatment **(B)**, and CPI values **(C)**. **(D)** Percentage of TC associated with each cluster relative to the total number of TC. The dashed box covers the ICS having more TC around. The parameters to choose TC are given in Supplementary Table 1. **(E)** Visualization of cluster centers with color consistent with **(A)**. Each percentage on the line show the percentage of TC between two clusters relative to the total number of cells. Arrowed solid line shows the main transition trajectory. **(F)** Violin plot of pseudotime value of each cell vs the collection time points. Each dot represents a cell colored by collection time points. The circle displays the mean and vertical line shows the interquartile ranges.

We applied a similar analysis to EMT induced by TNF and also identified four clusters with two ICSs (Supplementary Figure 3A and Figure 3A). Similar to the case of EGF induction, cells collected at different time points were mixed up in different clusters (Figure 3B). After selecting cluster C3 as the E state based on fewer TCs around (Figures 3C,D) and expression levels of canonical epithelial and mesenchymal marker genes (Supplementary Figures 3B,C), the most probable transition trajectories were revealed (Figure 3E). Based on the GO analysis of the top marker genes (Supplementary Figure 3D), the two ICSs were different states (Supplementary Figure 3E). The I1 state was related to signaling and locomotion indicating the communications with other cells and sharing mesenchymal features.

Similar to EMT induced by EGF, the average pseudotime values slightly increased across time points with high variance within each time point, thus suggesting the heterogeneity of cells undergoing EMT (Figure 3F). Therefore, EMT induced by TNF was also found to be an asynchronous process.

### Context-Specific Cellular Communications With Underlying Gene Regulations in TGF-β Signaling

**FIGURE 4:**
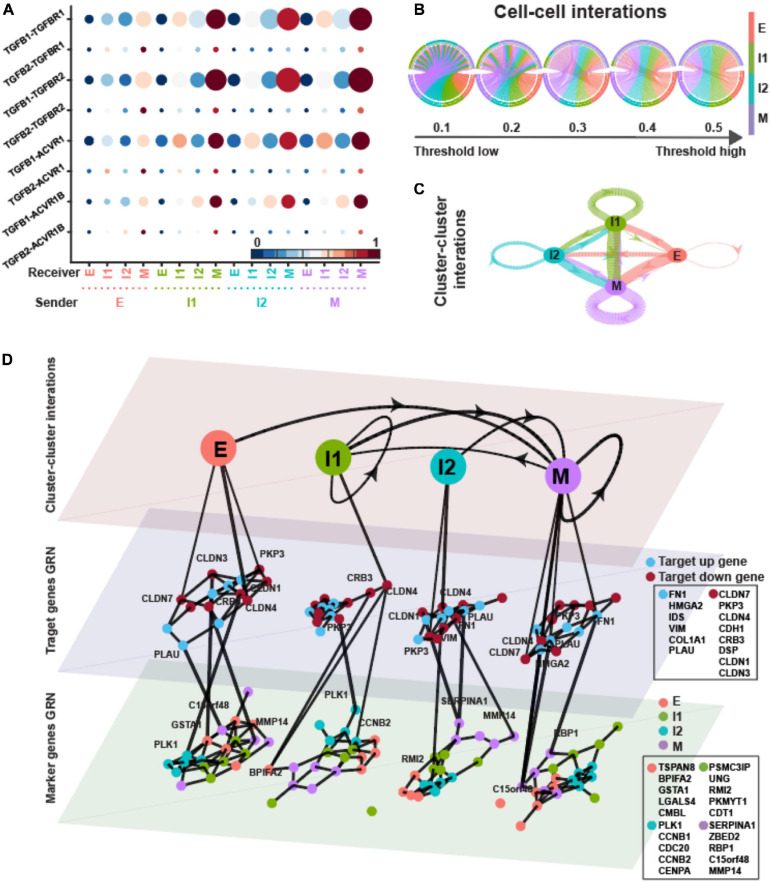
TGFB pathway on OVCA420 cancer cell line undergoing EMT induced by TGFB1. **(A)** Visualization of signaling probability scores of Ligand-Receptor pairs and their downstream signaling components. Dot size represents the number of averaged cells with non-zero probability scores between clusters. Dot color represents the signaling probability scores. **(B)** Circos plot of intercellular network on the top ten ligand-producing and top ten receptor-bearing cells from every cluster. The upper hemisphere of the plot shows receptor-bearing cells. The chords of the plot are colored by the ligand-producing cells in the lower hemisphere. The directed edges from the lower hemisphere to the upper hemisphere represent the probabilities of signaling between cells. The probabilities of signaling between cells above the thresholds are presented. **(C)** Intercluster network. The widths of edges are proportional to the signaling probability scores between clusters. The directed edges are colored by the ligand-producing clusters. **(D)** Multilayer network. The first layer shows the intercluster network as in **(C)** but with higher signaling probabilities greater than 0.5. Second and third layers show gene regulatory networks of target genes and top marker genes of clusters, respectively, using the PIDC algorithm. The target up (down) genes are the up-regulated (down-regulated) target genes of TGF-β signaling. Each dot represents a gene colored by its type. Graph edges indicate the top interactions and the length of the edge is inversely proportional to the interaction strength between genes. The link between first and second layer indicates the target gene are higher expressed within the cluster. The link between second and third layer indicates the strong interaction strength between target and marker genes. The widths of links between layers are proportional to the interaction strength. The ligands, receptors and target genes are given in Supplementary Table 3.

Transforming growth factor-β is a strong promoter of EMT (Hao et al., 2019). TGF-β ligands are not exclusively provided as an external EMT-inducing signal, but can also be secreted by cells, thus raising the possibility of cell–cell communication and EMT driven by intercellular signaling. In order to determine the possible role of TGF-β signaling in EMT, we assembled *in silico* ligand–receptor interaction pairs to explore the crosstalk between ICSs and E/M states. We applied SoptSC (Wang S. et al., 2019) to the expression matrix with inferred states and calculated the signaling probability of each ligand–receptor pair and their downstream targets between pairs of cells. Finally, averaging these pairwise signaling probabilities within each EMT state provides a snapshot of how cells tend to communicate based on their degree of EMT progression (Figures 4A–C).

In Figure 4B, the directed edges from lower hemisphere to upper hemisphere were inferred between cells where a high probability of signaling was predicted according to the expressions of ligands in a “sender” (lower hemisphere in the figure) cell and the appropriate expressions of cognate receptors and target genes in a “receiver” cell (upper hemisphere in the figure). The large proportion of M state behaving as “receiver” with high signaling probabilities suggests that the M state played a dominant role as receiver in TGF-β signaling. All the four states behaved as “sender” in TGF-β signaling.

The cluster–cluster signaling network was then constructed based on the average cell–cell signaling within each cluster (Figure 4C). We used strength, closeness, and pagerank as metrics to measure node centrality in the signaling network so that we can quantify the centralities of states in TGF-β signaling. Strength is defined as the sum over weights of the adjacent edges for a given node. Closeness of a node is the inverse of the average length of the shortest path to/from all the other nodes. Pagerank is proportional to the average time spent at a given node during all random walks; therefore, we interpret a high pagerank score as an indication that a node serves as a signaling hub in the network. The pagerank centrality of I1 and that of M were higher, thus showing the signaling hub potential (Supplementary Table 2). The I1 and M states had higher in-strength and lower in-closeness indicating that they behaved more like receivers (Supplementary Table 2).

To explore the change of the GRNs underlying TGF-β signaling with respect to EMT progress, we applied PIDC (Chan et al., 2017), an algorithm using partial information decomposition to identify GRNs, to the gene expression matrix of target genes and marker genes inferred by QuanTC within each state. In the dataset induced by TGFB1, the first layer of the multilayer network showed the cluster–cluster interactions as in Figure 4C but with only higher signaling probabilities greater than 0.5 (Figure 4D, top layer). The widths of the directed lines were proportional to the signaling probabilities. The central and bottom layers displayed the GRNs of target genes and marker genes within each state, respectively. The interactions between genes within each state were shown by the edges with lengths inversely proportional to the correlations between genes.

Based on the average correlations between target genes of TGF-β signaling and marker genes (Supplementary Figure 1F), both the up-regulated target genes and down-regulated target genes had stronger interactions with marker genes within E and M states. The up-regulated target genes always had largest correlations with marker genes of M stats, whereas the down-regulated target genes had relatively larger correlations with E marker within only E and M states.

**FIGURE 5:**
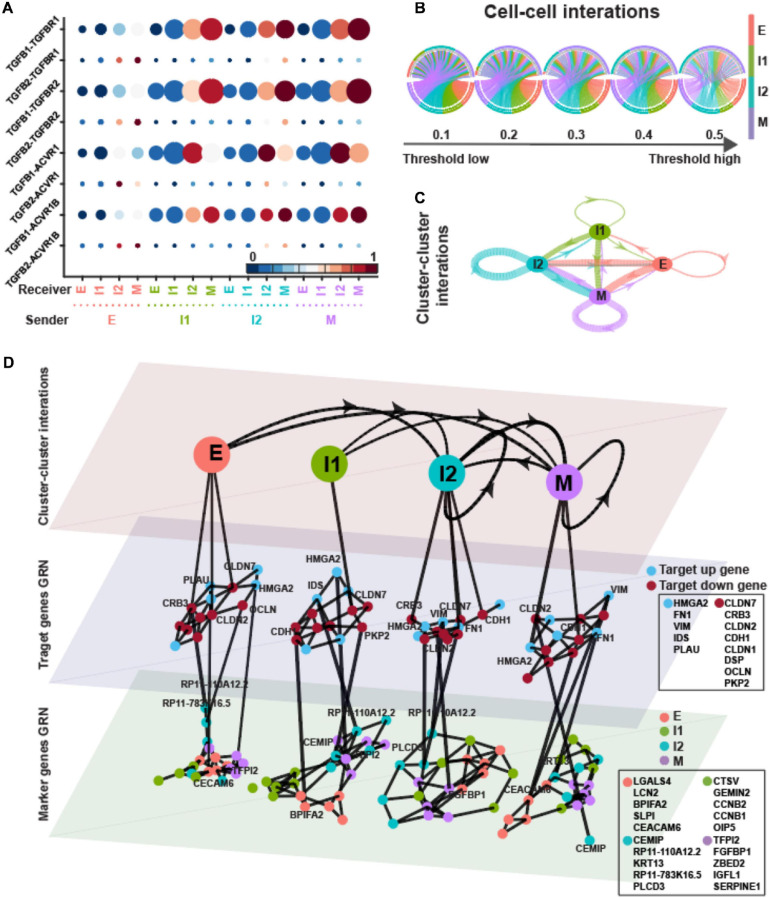
TGFB pathway on OVCA420 cancer cell line undergoing EMT induced by EGF. **(A)** Visualization of signaling probability scores of Ligand-Receptor pairs and their downstream signaling components. Dot size represents the number of averaged cells with non-zero probability scores between clusters. Dot color represents the signaling probability scores. Dot color represents the signaling probability scores. **(B)** Circos plot of intercellular network on the top ten ligand-producing and top ten receptor-bearing cells from every cluster. The upper hemisphere of the plot shows receptor-bearing cells. The chords of the plot are colored by the ligand-producing cells in the lower hemisphere. The directed edges from the lower hemisphere to the upper hemisphere represent the probabilities of signaling between cells. The probabilities of signaling between cells above the thresholds are presented. **(C)** Intercluster network. The widths of edges are proportional to the signaling probability scores between clusters. The directed edges are colored by the ligand-producing clusters. **(D)** Multilayer network. The first layer shows the intercluster network as in **(C)** but with higher signaling probabilities greater than 0.5. Second and third layers show gene regulatory networks of target genes and top marker genes of clusters, respectively, using the PIDC algorithm. The target up (down) genes are the up-regulated (down-regulated) target genes of TGF-β signaling. Each dot represents a gene colored by its type. Graph edges indicate the top interactions and the length of the edge is inversely proportional to the interaction strength between genes. The link between first and second layer indicates the target gene are higher expressed within the cluster. The link between second and third layer indicates the strong interaction strength between target and marker genes. The widths of links between layers are proportional to the interaction strength. The ligands, receptors and target genes are given in Supplementary Table 3.

In the dataset of EMT induced by EGF, the average TGF-β signaling probabilities suggest that I2 and M states played important roles as receivers, whereas all four states shared similar importance as senders (Figures 5A–C). Compared to EMT induced by TGFB1, the pagerank centrality of I2, instead of I1, and M states were higher (Supplementary Table 2).

In the multilayer network, the highly varied target genes were quite similar to EMT induced by TGFB1 (Figures 4, 5D). The up-regulated target genes were the same except missing COL1A1, and five out of the eight down-regulated target genes were the same as in Figure 4D. However, the top five marker genes of each state varied between the two treatments. Only LGALS4, BPIFA2, and ZBED2 shared marker genes of E and M states. CCNB1 and CCNB2, used to be I2 markers, were I1 markers for EMT induced by EGF.

The average correlations between target genes and marker genes were stronger within the I1 state (Supplementary Figure 2F). The up-regulated target genes did not always have largest correlations with marker genes of M state but still with relatively large correlations. The down-regulated target genes had stronger correlations with E markers except in the M state.

**FIGURE 6:**
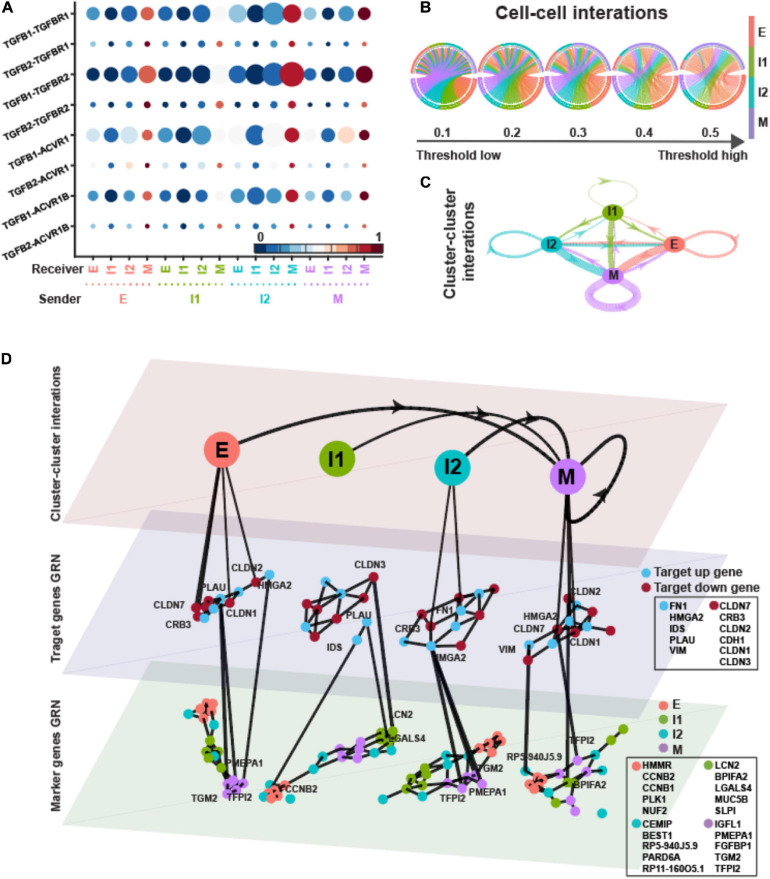
TGFB pathway on OVCA420 cancer cell line undergoing EMT induced by TNF. **(A)** Visualization of signaling probability scores of Ligand-Receptor pairs and their downstream signaling components. Dot size represents the number of averaged cells with non-zero probability scores between clusters. Dot color represents the signaling probability scores. **(B)** Circos plot of intercellular network on the top ten ligand-producing and top ten receptor-bearing cells from every cluster. The upper hemisphere of the plot shows receptor-bearing cells. The chords of the plot are colored by the ligand-producing cells in the lower hemisphere. The directed edges from the lower hemisphere to the upper hemisphere represent the probabilities of signaling between cells. The probabilities of signaling between cells above the thresholds are presented. **(C)** Intercluster network. The widths of edges are proportional to the signaling probability scores between clusters. The directed edges are colored by the ligand-producing clusters. **(D)** Multilayer network. The first layer shows the intercluster network as in **(C)** but with higher signaling probabilities greater than 0.5. Second and third layers show gene regulatory networks of target genes and top marker genes of clusters, respectively, using the PIDC algorithm. The target up (down) genes are the up-regulated (down-regulated) target genes of TGF-β signaling. Each dot represents a gene colored by its type. Graph edges indicate the top interactions and the length of the edge is inversely proportional to the interaction strength between genes. The link between first and second layer indicates the target gene are higher expressed within the cluster. The link between second and third layer indicates the strong interaction strength between target and marker genes. The widths of links between layers are proportional to the interaction strength. The ligands, receptors and target genes are given in Supplementary Table 3.

In the dataset of EMT induced by TNF, the different EMT states seemed to have similar importance as sender in TGF-β signaling (Figures 6A–C). The E and M states behaved as the main receivers. The M state had higher pagerank value showing the potential of signaling hub (Supplementary Table 2).

In the multilayer network, the varied up-regulated target genes were the subset of the genes in EMT induced by EGF except having CLDN3, and the down-regulated target genes were the subset of those genes in EMT induced by TGFB1 (Figures 4–6D). More than half of the marker genes of E, I1, and M states were the same as in EMT induced by EGF, suggesting the similarity of the EMT under the two treatments.

The target genes and marker genes had higher correlations within the I2 state (Supplementary Figure 3F). The up-regulated target genes always had relatively large correlations with marker genes of M state. The down-regulated target genes had stronger correlations with E markers except in the I2 state.

Overall, the M state and part of the ICSs behaved as the signaling hub in the TGF-β signaling of EMT under three different treatments (Figures 4–6). The M state was the main receiver in OVCA420 under three treatments with lowest in-closeness (Supplementary Table 2), while the underlying GRNs changed between different treatments and along EMT progress. Besides, the top marker genes of different EMT states were quite different among the EMT induced by different treatments, all suggesting the context-specific regulation of GRNs during EMT.

### Dominant Role of ICSs *in vivo* During TGF-β Signaling

Finally, we compare the results obtained for OVCA420 cells with *in vivo* data from a skin SCC mouse model to seek whether the defining traits of EMT dynamics are conserved or context-specific. In the original study, a total of six distinct cell populations were identified based on differential expression of cell surface markers (CD106, CD61, and CD51), including four transition states (Pastushenko et al., 2018).

**FIGURE 7:**
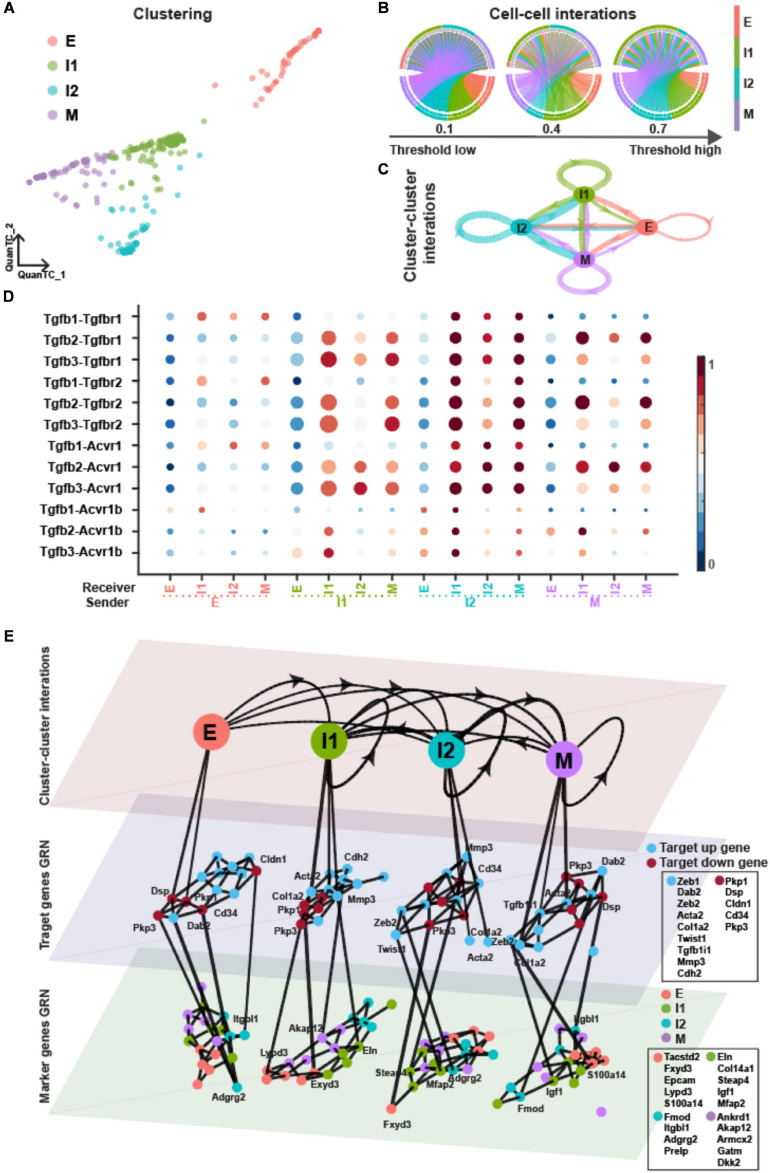
TGF-β pathway on EMT in SCC dataset. **(A)** Visualization of cells using QuanTC. Each circle represents a cell colored by corresponding cell state. **(B)** Circos plot of intercellular network on the top ten ligand-producing and top ten receptor-bearing cells from every cluster. The upper hemisphere of the plot shows receptor-bearing cells. The chords of the plot are colored by the ligand-producing cells in the lower hemisphere. The directed edges from the lower hemisphere to the upper hemisphere represent the probabilities of signaling between cells. The probabilities of signaling between cells above the thresholds are presented. **(C)** Intercluster network. The widths of edges are proportional to the signaling probability scores between clusters. The directed edges are colored by the ligand-producing clusters. **(D)** Visualization of signaling probability scores of Ligand-Receptor pairs and their downstream signaling components. Dot size represents the number of averaged cells with non-zero probability scores between clusters. Dot color represents the signaling probability scores. **(E)** Multilayer network. The first layer shows the intercluster network as in **(C)** but with higher signaling probabilities greater than 0.5. Second and third layers show gene regulatory networks of target genes and top marker genes of clusters, respectively, using the PIDC algorithm. The target up (down) genes are the up-regulated (down-regulated) target genes of TGF-β signaling. Each dot represents a gene colored by its type. Graph edges indicate the top interactions and the length of the edge is inversely proportional to the interaction strength between genes. The link between first and second layer indicates the target gene are higher expressed within the cluster. The link between second and third layer indicates the strong interaction strength between target and marker genes. The widths of links between layers are proportional to the interaction strength. The ligands, receptors and target genes are given in Supplementary Table 3.

In our previous work (Sha et al., 2020), we identified a total of four EMT states (Supplementary Figure 4A and Figure 7A) when applying QuanTC unsupervised clustering (Pastushenko et al., 2018). There were two ICSs displaying biological processes including cell–cell adhesion and cell migration indicating hybrid epithelial/mesenchymal features (Supplementary Figure 4B).

Compared to the OVCA420 cancer cell line undergoing EMT, the ICSs in SCC had higher probabilities of signaling and played the even more dominant role of cell–cell and cluster–cluster interactions during TGF-β signaling (Figures 7B–D). The ICSs, especially the I1 state, had higher Pagerank scores and served as the signaling hub (Supplementary Table 2). Both ICSs had lower out-closeness score, indicating that they played the dominant role as the sender in TGF-β signaling. While the M state had by far the higher pagerank score in the three OVCA420 datasets, the pagerank score of the M state in SCC was comparable to those of I1 and I2. Consistently, in the original study, the mesenchymal SCC exhibited a “quasi-mesenchymal” phenotype, which was more similar to intermediate state, instead of a fully mesenchymal phenotype (Pastushenko et al., 2018).

The highly varied target genes and marker genes of each state shared no similarity to the OVCA420 cancer line (Figure 7E). The target genes had strong associations with inferred marker genes within E and I1 states (Supplementary Figure 4C). It suggests that EMT varies both between mouse vs human, and *in vitro* vs *in vivo*.

## Materials and Methods

### scRNA-Seq Data Clustering and Transition Trajectory Reconstruction

QuanTC was used to perform clustering and transition trajectory reconstruction. QuanTC can simultaneously detect the ICSs and construct transition trajectories via quantifying the CPI (Sha et al., 2020). The cells with higher CPI values are considered to be transitioning between clusters and are identified as TCs. Via non-negative matrix factorization, QuanTC calculates the probabilities of a given cell belonging to the identified clusters. Cells are projected to a low-dimensional space based on a probabilistic regularized embedding. The transition trajectories are then inferred by summing the cluster-to-cluster transition probabilities that are calculated from cell-to-cluster probabilities and TCs between clusters. The transition genes and marker genes of clusters are obtained through factorizing the gene expression matrix as product of cell-to-cluster probabilities and likelihoods of genes uniquely marking each cluster. In the first step of QuanTC, we applied two additional considerations when choosing the number of identified clusters. First, we know from the original experiment that cells undergo EMT (i.e., there is at least one E state and one M state); furthermore, given that we seek to study ICSs during EMT, we search for at least three total states.

#### Preprocessing

Single cells with less than 95% expressed genes among all detected genes were considered as low-quality cells and were filtered. Top 3,000 bimodal distributed genes were selected by QuanTC with default parameters to do downstream analysis.

#### Clustering

A total of 3,000 selected genes and 558 cells of OVCA420 induced by TGFB1, 1,137 cells of OVCA420 induced by EGF, and 1,856 cells of OVCA420 induced by TNF from day 0 to day 7 were retained for clustering. Consensus clustering via SC3 (Kiselev et al., 2017) was performed on the expression matrix to capture the cell–cell similarity. The clusters were defined based on symmetric non-negative factorization as wrapped in QuanTC.

#### Transition Trajectory

The beginning and end of EMT transition trajectory, E/M states, were identified based on the percentage of TCs around each cluster. The parameters to choose TCs were given in Supplementary Table 1. The clusters with fewer TCs around were considered as E/M states, whereas the rest clusters were considered as ICSs along EMT. The E/M states between the two clusters were then identified based on the canonical epithelial and mesenchymal marker genes. The potential transition trajectory was inferred according to the TCs between clusters using “traj” function wrapped in QuanTC. The pseudotime value of each cell was then computed by QuanTC based on the two most probable trajectories.

#### EMT Marker Genes

The marker genes and transition genes were defined using “markers” function wrapped in QuanTC.

#### GO Analysis

The analysis of GO biological processes was performed by Metascape (Zhou et al., 2019) on the top 50 markers genes of each ICS selected by QuanTC.

### Qualitatively Characterizing Cell–Cell Communications

SoptSC (Wang S. et al., 2019) was used on the datasets without gene filtering to calculate the probability matrix of signals being passed between cells and clusters. Signaling probabilities between cells are defined based on weighted co-expression of signaling pathway activity in sender–receiver cell pairs. With the input of ligand–receptor pairs and target genes (up-regulated or down-regulated in response to pathway activation), SoptSC computes signaling probabilities between sender cells (expressing ligands) and receiver cells (expressing receptors and exhibiting differential target genes activity). Intuitively, given a ligand–receptor pair for a specific signaling pathway, if the ligand is highly expressed in cell *i*, the cognate receptor is highly expressed in cell *j*, and the target gene activity in cell *j* suggests that the signaling pathway may have been activated in this cell, and then there is a chance that communication occurred between these two cells. The signaling passed from cell *i* to *j* for a given ligand–receptor pair is quantified by the signaling probability *P*_*i,j*_. For a set of ligand–receptor pairs, SoptSC considers the consensus signaling probabilities between cells by taking the average over all signaling probability matrices. The signaling probability passed from cluster *u* to cluster *v* is then given by $P_{u,v}=\frac{\sum_{i\in C_{u},j\in C_{v}}P_{i,j}}{\left|C_{u}\right|\,\left|C_{v}\right|}$, with |*C*_*u*_| representing the number of cells in cluster *u*.

The lists of ligands, receptors, and target genes were retrieved from previous studies (Wendt et al., 2009; Xu et al., 2009; Jin et al., 2020) and are given in Supplementary Table 3.

### Measuring Node Centrality

The centrality of a node (cluster) in cellular communication network is used to quantify its importance in the signaling. We used strength, closeness, and pagerank as metrics to measure node centrality. All these centralities were calculated with the package igraph 1.2.4 (Csardi and Nepusz, 2006).

Strength is one of the basic measures of centrality: it is measured by summing up the edge weights of the adjacent edges for a given node. Our inferred cluster–cluster communication networks are directed, so we calculated in-strength (incoming edges), and out-strength (outgoing edges). Closeness of a given node is defined by the inverse of the average length of the shortest path to/from all the other nodes. In-closeness measures the path to the node, whereas out-closeness measures the paths from the node. We used the normalized values to avoid biases based on the network size. Pagerank is proportional to the average time spent at a given node during all random walks. In the cluster–cluster communication networks, the clusters with high pagerank can be seen as the signaling hub.

### Multilayer Regulations of EMT

We utilized the multilayer network framework (Kivelä et al., 2014) to analyze and visualize the changes of complex hierarchical signaling and gene expression regulations in EMT across multiple scales.

Mathematically, the multilayer network can be expressed as the *M* = (*V*_*M*_,*E*_*M*_,*V*,**L**). Here, *V* denotes sets of all nodes in the network (as in the regular case), and $\text{L}={\left\{L_{a}\right\}}_{a=1}^{d}$ denotes *d* aspects of the network layers, with each aspect $L_{a}={\left\{L_{a}^{i}\right\}}_{i=1}^{k_{a}}$ contains *k*_*a*_ elementary layers. Denotes × as the Cartesian product of sets, and then the node–layer tuple set *V*_*M*_⊆*V*×*L*_1_×⋯×*L*_*d*_ represents all the feasible node–layer combinations in which a node is present in the corresponding layers. The edges set *E*_*M*_⊆*V*_*M*_×*V*_*M*_ denotes the weighted links across nodes and layers.

In our context, the nodes set *V* not only contains cell states $S={⋃}_{k=1}^{N_{c}}S_{k}$ along the EMT trajectories, with *N*_*c*_ denoting the number of cell states, but also contains target genes *T* of specified signal transduction pathway and marker genes *A* of each cell state. The layers **L** = {*L*_*H*_,*L*_*C*_} has two aspects: The hierarchy aspect $L_{H}=\left\{L_{H}^{1},L_{H}^{2},L_{H}^{3}\right\}$ represents the elementary layers of cell–cell communication $L_{H}^{1}$, target genes $L_{H}^{2}$, and marker genes $L_{H}^{3}$, respectively, and the cell states aspect $L_{C}={\left\{L_{C}^{k}\right\}}_{k=1}^{N_{c}}$ represents the EMT stages of E state, ICSs, and M state ordered by pseudotime of QuanTC, as we are interested in constructing cell-state–specific regulatory relations. For simplicity, we denote the node–layer tuples in EMT as $V_{M}=\left\{\left(S,L_{H}^{1},\cdot \right),\left(T,L_{H}^{2},\cdot \right),\left(A,L_{H}^{3},\cdot \right)\right\}\subseteq V\times L_{H}\times L_{C}$, representing the hierarchical regulation structures at different stages. For instance, $\left(A,L_{H}^{3},L_{C}^{1}\right)$ denotes the marker genes analyzed in the E state, while $\left(T,L_{H}^{2},L_{C}^{2}\right)$ represents the target genes considered in the first ICSs. We next specify how the edges *E*_*M*_ are constructed based on the *V*_*M*_.

#### The Edges Within Layer $\left(S,{\text{L}}_{H}^{1},\cdot \right)$

The first layer $L_{H}^{1}$ in hierarchy aspect displays the cluster–cluster interactions of intercellular communication, where the aligned nodes show the different EMT states/clusters. Using the notations above, $\left(S,L_{H}^{1},L_{C}^{k}\right)$ contains only one node for each *k*, representing the cell state *S*_*k*_. The weights for the directed edges to connect $\left(S,L_{H}^{1},L_{C}^{i}\right)$ and $\left(S,L_{H}^{1},L_{C}^{j}\right)$ are the cluster–cluster interactions between state *S*_*i*_ and state *S*_*j*_ computed by SoptSC above threshold 0.7.

#### The Edges Within Layer $\left(T,{\text{L}}_{H}^{2},\cdot \right)$

The second layer $L_{H}^{2}$ demonstrates the state-specific interactions among target genes at different stages. The target genes *T* are the intersection of the list of target genes and the top 3,000 selected informative genes. Given the stage $L_{C}^{k}$, the weighted edges between target gene pair $\left(T_{X},L_{H}^{2},L_{C}^{k}\right)$ and $\left(T_{Y},L_{H}^{2},L_{C}^{k}\right)$ were constructed by PIDC algorithm (Chan et al., 2017) using partial information decomposition, only with the cells in cluster *S*_*k*_. The input to PIDC is an expression matrix with cells from *S*_*k*_, and the confidence of an edge between a pair of genes is given by *c* = *F*_*X*_(*U*_*X*,*Y*_) + *F*_*Y*_(*U*_*X*,*Y*_) where *F*_*X*_(*U*) is the cumulative distribution function of all the proportional unique contribution scores involving gene *X*. The top 30% weights were used to embed the inferred network in $\left(T,L_{H}^{2},L_{C}^{k}\right)$ using “graph” function in MATLAB based on spectral layout (Koren, 2005). The weights were normalized with max 2 to be comparable with other datasets.

#### The Edges Within Layer $\left(A,{\text{L}}_{H}^{3},\cdot \right)$

The third layer $L_{H}^{3}$ demonstrates the state-specific interactions among marker genes at different stages. The marker genes selected were identical for $\left(A,L_{H}^{3},L_{C}^{k}\right)$ with respect to the choice of *k*, which represent the union of top five marker genes in each cluster inferred by QuanTC. The edges between marker genes are state-specific for each cell-state layer $L_{C}^{k}$, using the same strategy as for the target genes described above.

#### The Edges Connecting Layer $\left(S,L_{H}^{1},\cdot \right)$ and $\left(T,L_{H}^{2},\cdot \right)$

These edges quantify the expression of target genes within different states during EMT. The weights for the edges between $\left(S,L_{H}^{1},L_{C}^{k}\right)$ and $\left(T,L_{H}^{2},L_{C}^{k}\right)$ are the mean expression levels of target genes within cell state *S*_*k*_, and top 20% weights were shown.

#### The Edges Connecting Layer $\left(T,L_{H}^{2},\cdot \right)$ and $\left(A,L_{H}^{3},\cdot \right)$

These edges display the regulatory interactions from target genes to marker genes within different states during EMT. The weights for the edges between $\left(T,L_{H}^{2},L_{C}^{k}\right)$ and $\left(A,L_{H}^{3},L_{C}^{k}\right)$ were inferred by PIDC within cell state *S*_*k*_, and top 1.5% weights were shown.

## Discussion

In this study, we have developed an approach combining unsupervised learning, multivariate information theory, and multilayer network approach to uncover the complex cellular crosstalk and the underlying gene regulatory relationship of EMT from scRNA-seq data.

We started with trajectory reconstruction on the time-series datasets of an OVCA420 cancer cell line undergoing EMT induced by three different external signal (TGFB1, EGF, and TNF) and uncovered the existence of multiple ICSs displaying hybrid epithelial and mesenchymal features. Analysis of scRNA-seq previously demonstrated that EMT induction by TGFB1, EGF, and TNF is carried by context-specific signaling pathways (Cook and Vanderhyden, 2020). Here, we show striking differences in the EMT population dynamics as well. While EMT induced by TGFB1 is synchronous, EGF and TNF induce asynchronous transitions because cells collected at different time points spread all over different clusters. These differences at the cell population level could be explained by the signaling complexity and modularity in response to different EMT inducers. TNF can activate nuclear factor κB (NF-κB) signaling, which in turn crosstalks with several transduction pathways and induces responses to inflammation (Hayden and Ghosh, 2014). TNF–NF-κB signaling has also been proposed as a stability factor for hybrid E/M phenotypes, thus potentially resisting a complete EMT in TNF-induced EMT (Bocci et al., 2019). Similarly, EGF regulation of EMT is not direct, but rather relies on several intermediate signaling steps that could hamper a synchronized transition (Kang et al., 2013). Certainly, future efforts focusing on integrating high-throughput data analysis with *in silico* modeling of the underlying regulatory circuitry will help validate or falsify these hypotheses.

To clarify how cells in different EMT states contribute to cell–cell signaling, we subsequently constructed multilayer networks displaying the TGF-β signaling communication between cells in different EMT states and the underlying GRN that regulates EMT at different EMT stages. We found that ICSs serve as signaling hubs of cell–cell communication, as well as the context-specific response of TGF-β under different treatments. In other words, cells in intermediate EMT states can send and receive inputs from other cells through TGF-β signaling, potentially inducing EMT in their neighbors. Therefore, both cell autonomous TGFB1 induction and intercellular TGFB signaling could contribute to EMT. Future experiments controlling conditional knockouts of TGFB ligands could validate this prediction and quantify the role played by cell–cell communication in EMT. These observations also raise an interesting parallel with Notch signaling, another master regulator of cell–cell communication (Bray, 2016). Signaling through the Notch-Jagged pathway between cancer cells in intermediate EMT states has been proposed as a mechanism that (i) stabilizes intermediate EMT states and (ii) further induces “partial EMT” in other cells (Bocci et al., 2017; Jolly et al., 2017). Our analysis on *in vivo* dataset also suggests that ICS plays the more dominant role in the TGF-β signaling communication.

The core gene circuits for EMT are known to involve multiple molecular components and interactions (Jia et al., 2017; Tian et al., 2019; Yang et al., 2020), providing mechanisms of the EMT transition process (Jolly and Levine, 2017). Recent time-series scRNA-seq data suggest that EMT is indeed highly context-specific (Cook and Vanderhyden, 2020), calling for the need of inferring EMT regulation circuits from a data-driven approach (Tanaka and Ogishima, 2015; Ramirez et al., 2020). Previous works have constructed the GRN of EMT based on the combination of prior knowledge, transcription factor predictions, and model validations from single-cell datasets (Ramirez et al., 2020). Here we have incorporated the intercellular communications in the context of analyzing TCs and ICSs to inspect the dynamical change of regulation interactions along the EMT spectrum.

Our analysis reveals that ICS plays the crucial role in not only interchanging information with both pure epithelial and mesenchymal states, but also communicating with other cells in ICSs during EMT. Previously, the role of ICSs has been studied for tumor metastasis (Jolly et al., 2015) and analyzed through the emergent dynamical properties such as signal adaptation, noise attenuation, and population transition (Ta et al., 2016; Sha et al., 2019; Goetz et al., 2020). Taken together, the EMT cell lineage models with ICS-mediated feedback through cell–cell communications (Lander et al., 2009; Lo et al., 2009) could be further developed to explore the non-linear effects on different cell populations (Jia W. et al., 2019).

The integrative analysis here is a general approach and can be applied to other cell-state transition processes beyond EMT. In particular, the multiplayer gene regulatory and intercellular network provides a multiscale framework to simultaneously explore the cellular communications, the underlying gene regulations, and dynamics of GRNs along transitions. By incorporating additional layers of different transduction elements beyond TGF-β (Jin et al., 2020) and associated transcription factors, one can investigate the more complex regulation processes, such as signal crosstalk and corporation of multiple pathways (Xing and Tian, 2019). In addition, the inclusion of spatial information layer may also facilitate the accuracy of intercellular communication analysis (Cang and Nie, 2020).

Overall, our study provides an initial attempt to investigate the multiscale interactions of intercellular communications and gene expression regulations during the dynamical process of cell-fate determination.

## Data Availability Statement

Publicly available datasets were analyzed in this study. These datasets can be found here: SCC (GEO: GSE110357) and OVCA420 cancer cell line (GEO: GSE147405) datasets downloaded from the Gene Expression Omnibus.

## Author Contributions

QN, PZ, and YS conceived the study. YS implemented the algorithm and wrote the codes. YS, SW, and FB performed data analysis. YS, FB, PZ, and QN wrote the manuscript with the help from all the authors. QN and PZ supervised the research. All authors approved the manuscript.

## Conflict of Interest

The authors declare that the research was conducted in the absence of any commercial or financial relationships that could be construed as a potential conflict of interest.
