# Supplementary material for: Inference of Intercellular Communications and Multilayer Gene-Regulations of Epithelial–Mesenchymal Transition From Single-Cell Transcriptomic Data
Source: Front Genet. 2021 Jan 8;11:604585. doi: 10.3389/fgene.2020.604585 (PMC7820899; doi:10.3389/fgene.2020.604585)
Supplement: Supplementary file 1 [file Data_Sheet_1.PDF]

Supplementary Material

1 Supplementary Figures and Tables

1.1 Supplementary Figures

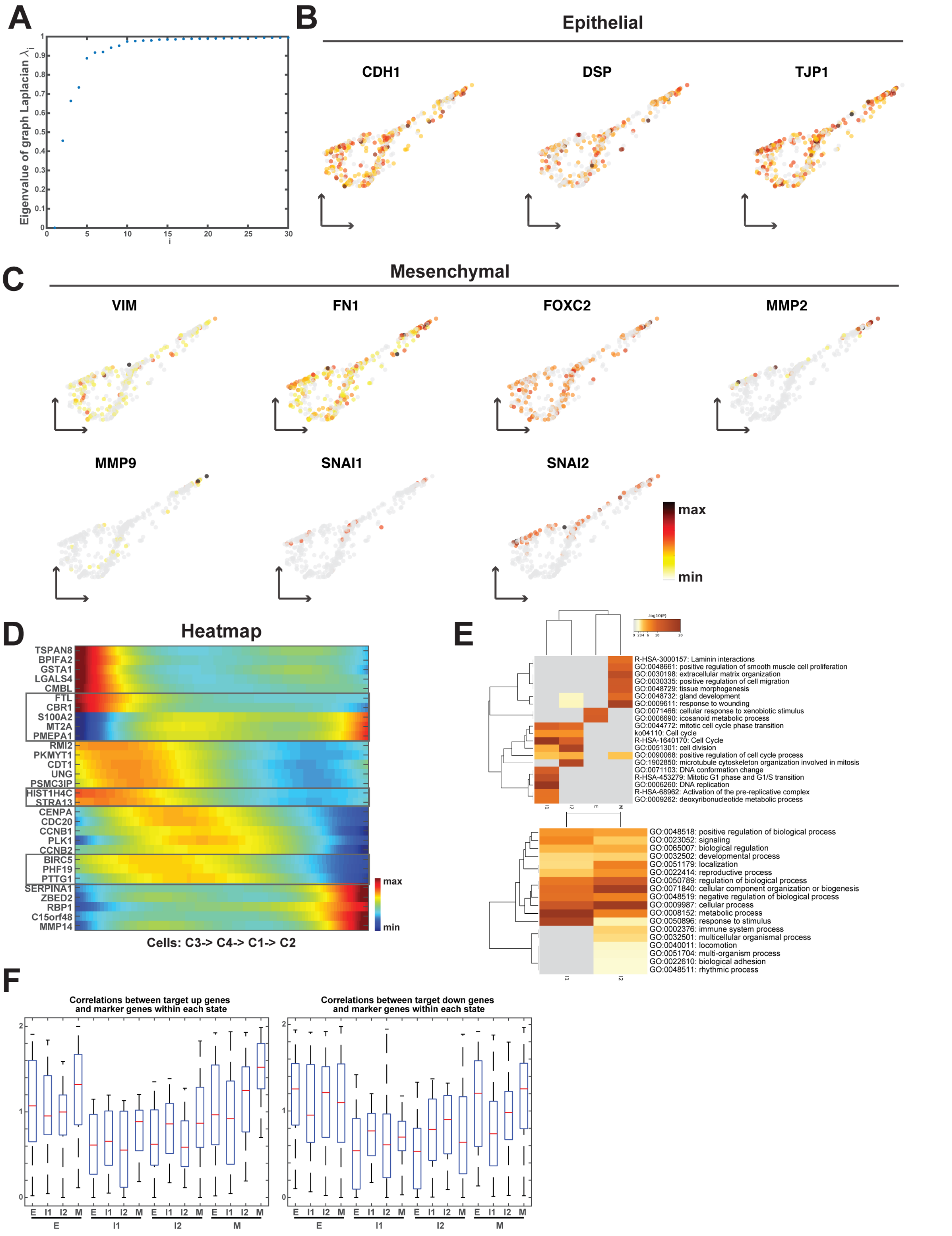

Supplementary Figure 1. OVCA420 cancer cell line undergoing EMT induced by TGFB1. (A) The first 30 sorted eigenvalues of the graph Laplacian of the cell-cell similarity matrix from consensus

clustering. (B-C) Dimensionality reduction of the dataset by QuanTC coloring for known epithelial genes (B) and mesenchymal genes (C). (D) Heatmap of normalized expression of marker genes and transition genes. Columns represent cells ordered along the transition trajectory and rows represent genes. Coloring represents the normalized expression value of each gene. (E) The top-level gene ontology biological processes analyzed by Metascape of the marker genes of all cell states and ICS respectively. (F) Boxplot of the correlations between target genes and marker genes from Fig. 4D within each state. The central red mark indicates the median, and the bottom and top edges of the box indicate the 25th and 75th percentiles, respectively. The whiskers extend to the most extreme data points.

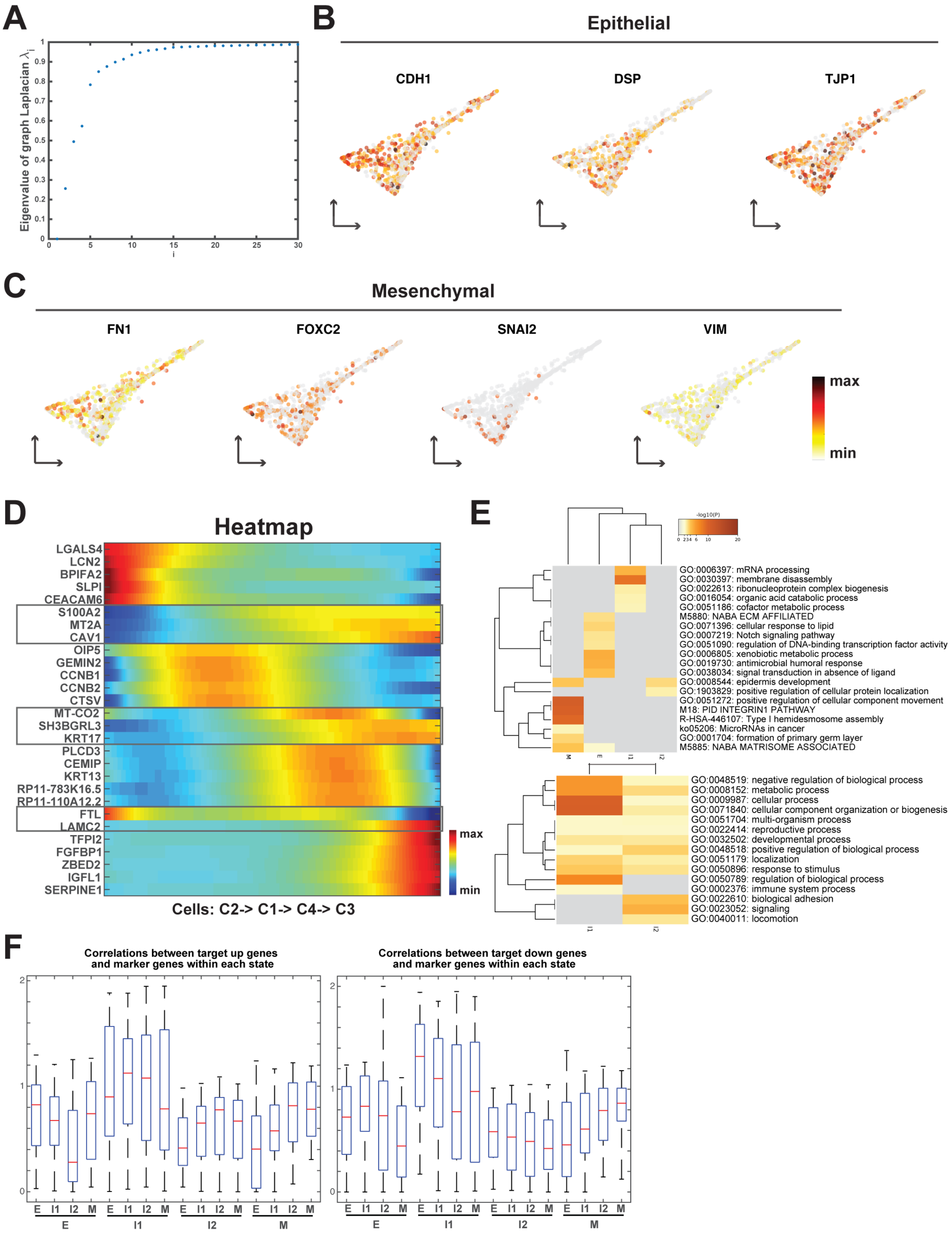

Supplementary Figure 2. OVCA420 cancer cell line undergoing EMT induced by EGF. (A) The first 30 sorted eigenvalues of the graph Laplacian of the cell-cell similarity matrix from consensus

clustering. (B-C) Dimensionality reduction of the dataset by QuanTC coloring for known epithelial genes (B) and mesenchymal genes (C). (D) Heatmap of normalized expression of marker genes and transition genes. Columns represent cells ordered along the transition trajectory and rows represent genes. Coloring represents the normalized expression value of each gene. (E) The top-level gene ontology biological processes analyzed by Metascape of the marker genes of all cell states and ICS respectively. (F) Boxplot of the correlations between target genes and marker genes from Fig. 4D within each state. The central red mark indicates the median, and the bottom and top edges of the box indicate the 25th and 75th percentiles, respectively. The whiskers extend to the most extreme data points.

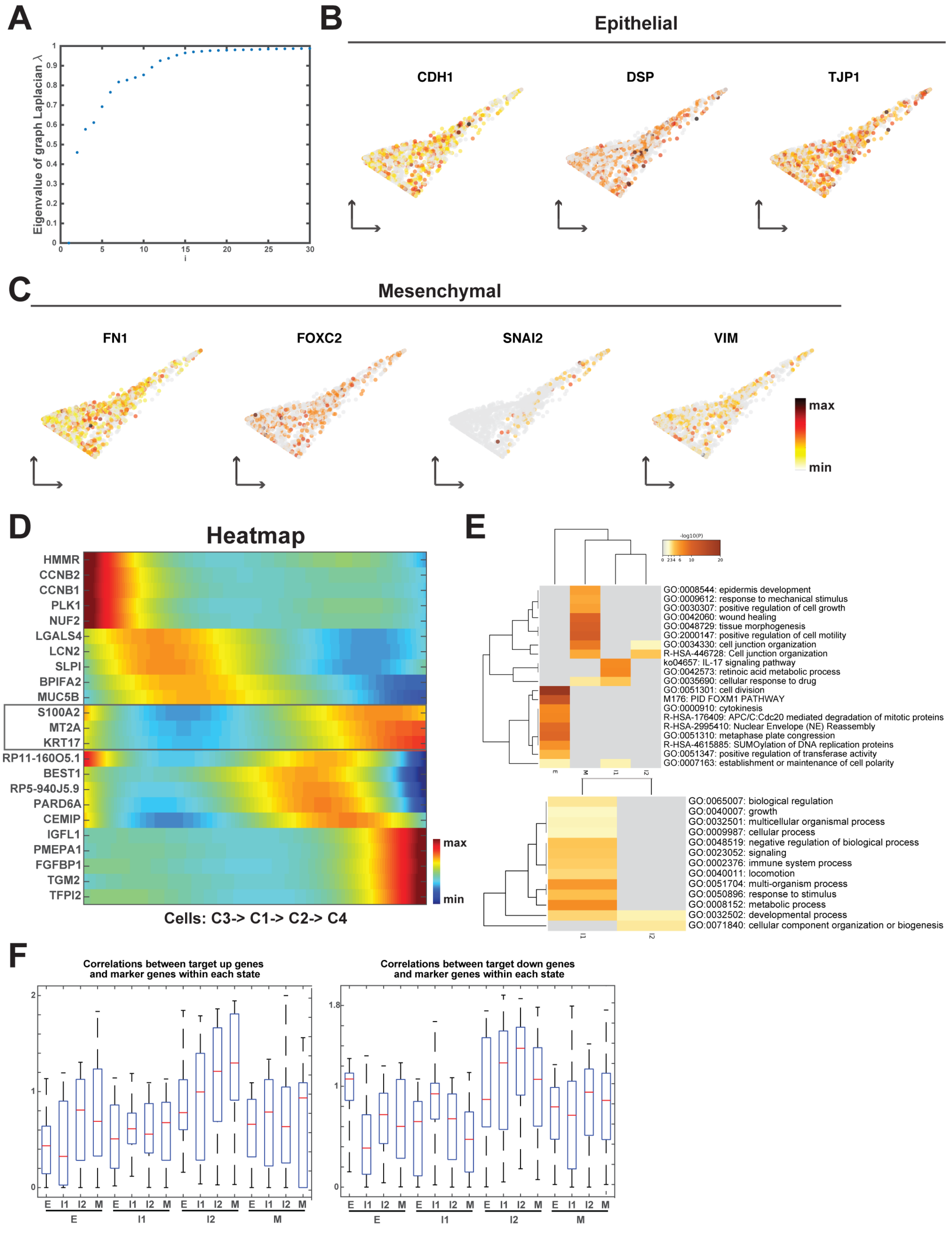

Supplementary Figure 3. OVCA420 cancer cell line undergoing EMT induced by TNF. (A) The first 30 sorted eigenvalues of the graph Laplacian of the cell-cell similarity matrix from consensus clustering. (B-C) Dimensionality reduction of the dataset by QuanTC coloring for known epithelial

genes (B) and mesenchymal genes (C). (D) Heatmap of normalized expression of marker genes and transition genes. Columns represent cells ordered along the transition trajectory and rows represent genes. Coloring represents the normalized expression value of each gene. (E) The top-level gene ontology biological processes analyzed by Metascape of the marker genes of all cell states and ICS respectively. (F) Boxplot of the correlations between target genes and marker genes from Fig. 4D within each state. The central red mark indicates the median, and the bottom and top edges of the box indicate the 25th and 75th percentiles, respectively. The whiskers extend to the most extreme data points.

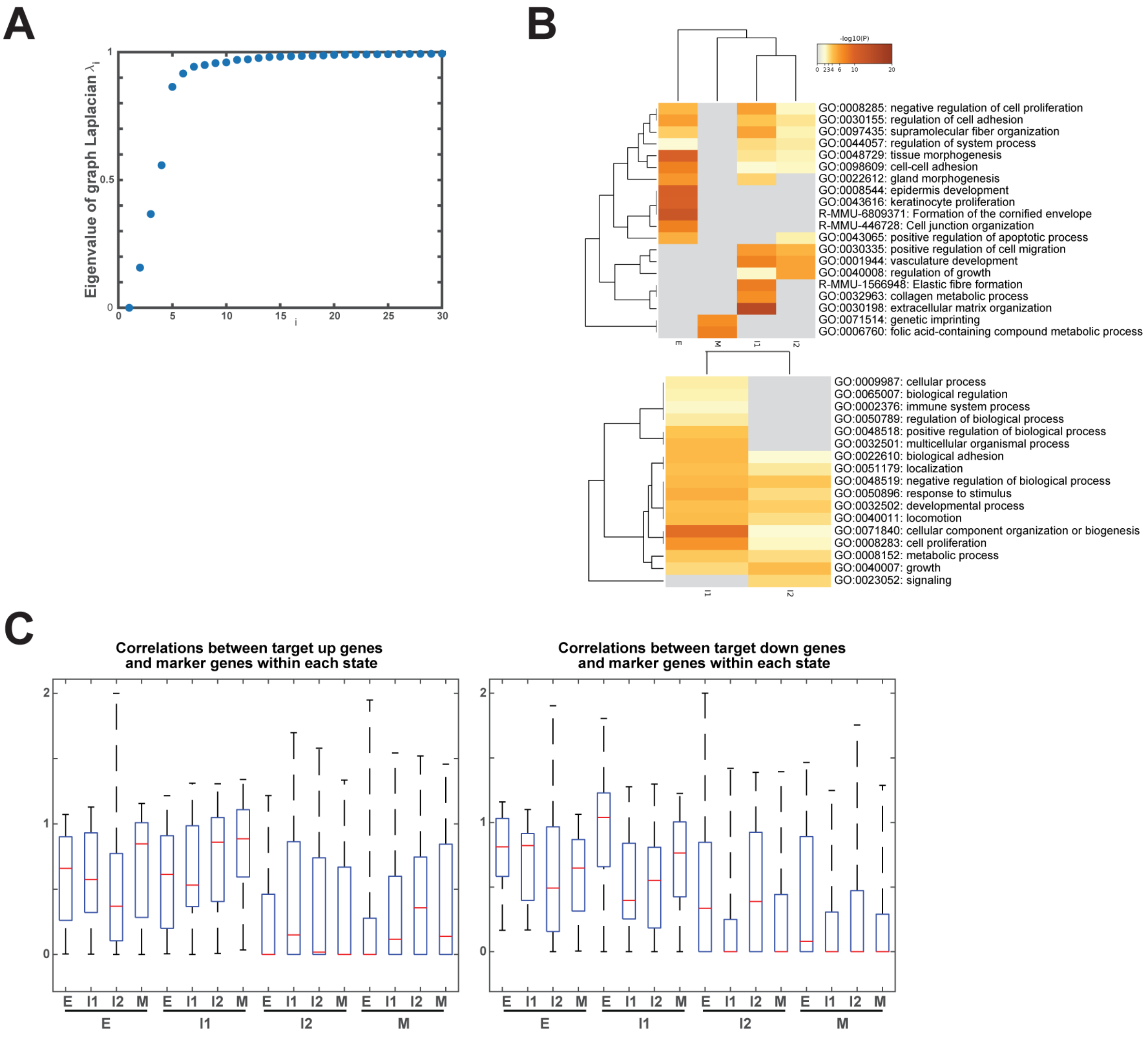

Supplementary Figure 4. (A) The first 30 sorted eigenvalues of the graph Laplacian of the cell-cell similarity matrix from consensus clustering. (B) The top-level gene ontology biological processes analyzed by Metascape of the marker genes of all cell states and ICS respectively. (C) Boxplot of the correlations between target genes and marker genes from Fig. 4D within each state. The central red mark indicates the median, and the bottom and top edges of the box indicate the 25th and 75th percentiles, respectively. The whiskers extend to the most extreme data points.

1.2 Supplementary Tables

Table S1. Thresholds of CPI values

	OVCA420 _TGFB1	OVCA420 _EGF	OVCA420 _TNF
Thresholds of CPI to select TC	0.4	0.55	0.4

Table S2. Measuring node centrality

		E	I1	I2	M
OVCA420_TGFB1	In-strength	0.1776162	1.9648623	1.2557214	3.4196665
	Out-strength	1.689800	1.993284	1.283902	1.850880
	In-closeness	22.565594	2.157533	2.940819	1.204396
	Out-closeness	1.823565	2.114316	2.861763	3.253483
	Pagerank	0.05957221	0.28235406	0.19392289	0.46415084
OVCA420_EGF	In-strength	0.5808987	0.6194308	3.0256635	3.3411261
	Out-strength	2.019406	1.465237	2.266148	1.816328
	In-closeness	7.040529	6.006635	1.415538	1.180988
	Out-closeness	1.608914	2.230063	2.206174	2.954360
	Pagerank	0.1027799	0.1071867	0.3774523	0.4125810
OVCA420_TNF	In-strength	1.1865127	0.3591376	1.1528117	3.6014175
	Out-strength	1.692995	1.192109	1.666992	1.747783

	In-closeness	3.458899	10.294901	3.538624	1.153217
	Out-closeness	2.183708	2.668141	2.202695	4.011858
	Pagerank	0.19736113	0.08600868	0.19305720	0.52357299
SCC	In-strength	1.506968	3.005718	2.501364	2.870468
	Out-strength	1.969614	2.411808	3.287782	2.215314
	In-closeness	2.488402	1.320666	1.796591	1.347659
	Out-closeness	1.798303	1.788193	1.221375	1.909698
	Pagerank	0.1670528	0.2960197	0.2525167	0.2844108

Table S3. TGFB pathway used for generating cell-to-cell signaling networks and cluster-to-cluster signaling networks

Ligand	Receptor	Target genes (up)	Target genes (down)
TGFB1	TGFBR1	FN1<br>VTN<br>CDH2<br>COL1A1 COL1A2<br>MMP2 MMP3 MMP9<br>TWIST1 TWIST2<br>IDS<br>ZEB1 ZEB2	OCLN CRB3 ESR1<br>CD34 CDH1 DSP<br>CLDN1 CLDN2<br>CLDN3 CLDN4<br>CLDN5 CLDN6<br>CLDN7 CLDN8<br>CLDN9 CLDN10<br>CLDN11 CLDN12
TGFB2	TGFBR1		
TGFB3	TGFBR1		
TGFB1	TGFBR2		
TGFB2	TGFBR2		
TGFB3	TGFBR2		
TGFB1	ACVR1		

<table>
<tr><td>TGFB2</td><td>ACVR1</td><td rowspan="8">SPARC
ITGA5 ITGB3
NCAM
VIM
ACTA2
PLAU
DAB2
HIC5
TGFB1I1
HMGA2</td><td rowspan="8">CLDN13 CLDN14
CLDN15 CLDN16
CLDN17 CLDN18
CLDN19 CLDN20
CLDN21 CLDN22
CLDN23
PKP1 PKP2 PKP3
CK5 CK14 CK8
CK18</td></tr>
<tr><td>TGFB3</td><td>ACVR1</td></tr>
<tr><td>TGFB1</td><td>ACVR1B</td></tr>
<tr><td>TGFB2</td><td>ACVR1B</td></tr>
<tr><td>TGFB3</td><td>ACVR1B</td></tr>
<tr><td>TGFB1</td><td>ACVR1C</td></tr>
<tr><td>TGFB2</td><td>ACVR1C</td></tr>
<tr><td>TGFB3</td><td>ACVR1C</td></tr>
</table>
